# Supplementary material for: Exploration of the impact of the COVID-19 pandemic on people with dementia and carers from black and minority ethnic groups
Source: BMJ Open. 2021 May 18;11(5):e050066. doi: 10.1136/bmjopen-2021-050066 (PMC8136797; doi:10.1136/bmjopen-2021-050066)
Supplement: Author's manuscript [file bmjopen-2021-050066.draft_revisions.pdf]

BMJ Open is committed to open peer review. As part of this commitment we make the peer review history of every article we publish publicly available.

When an article is published we post the peer reviewers' comments and the authors' responses online. We also post the versions of the paper that were used during peer review. These are the versions that the peer review comments apply to.

The versions of the paper that follow are the versions that were submitted during the peer review process. They are not the versions of record or the final published versions. They should not be cited or distributed as the published version of this manuscript.

BMJ Open is an open access journal and the full, final, typeset and author-corrected version of record of the manuscript is available on our site with no access controls, subscription charges or pay-per-view fees (http://bmjopen.bmj.com).

If you have any questions on BMJ Open's open peer review process please email
info.bmjopen@bmj.com

BMJ Open

An exploration of the impact of the Covid-19 pandemic on people with dementia and carers from Black and Minority Ethnic groups

Journal:	*BMJ Open*
Manuscript ID	bmjopen-2021-050066
Article Type:	Original research
Date Submitted by the Author:	11-Feb-2021

Complete List of Authors:	West, Emily; University College London,<br>Nair, Pushpa; University College London, Department of Primary Care and Population Health<br>Barrado-Martin, Yolanda; University College London, Department of Primary Care and Population Health<br>Walters, Kate; University College London, Primary Care and Population Health<br>Kupeli, Nuriye; Marie Curie Palliative Care Research Unit, UCL Division of Psychiatry<br>Sampson, Elizabeth; University College London, Division of Psychiatry<br>Davies, Nathan; UCL, Department of Primary Care and Population Health

Keywords:	Dementia < NEUROLOGY, Adult palliative care < PALLIATIVE CARE, PALLIATIVE CARE

SCHOLARONE™
Manuscripts

I, the Submitting Author has the right to grant and does grant on behalf of all authors of the Work (as defined in the below author licence), an exclusive licence and/or a non-exclusive licence for contributions from authors who are: i) UK Crown employees; ii) where BMJ has agreed a CC-BY licence shall apply, and/or iii) in accordance with the terms applicable for US Federal Government officers or employees acting as part of their official duties; on a worldwide, perpetual, irrevocable, royalty-free basis to BMJ Publishing Group Ltd ("BMJ") its licensees and where the relevant Journal is co-owned by BMJ to the co-owners of the Journal, to publish the Work in this journal and any other BMJ products and to exploit all rights, as set out in our licence.

The Submitting Author accepts and understands that any supply made under these terms is made by BMJ to the Submitting Author unless you are acting as an employee on behalf of your employer or a postgraduate student of an affiliated institution which is paying any applicable article publishing charge ("APC") for Open Access articles. Where the Submitting Author wishes to make the Work available on an Open Access basis (and intends to pay the relevant APC), the terms of reuse of such Open Access shall be governed by a Creative Commons licence – details of these licences and which Creative Commons licence will apply to this Work are set out in our licence referred to above.

Other than as permitted in any relevant BMJ Author's Self Archiving Policies, I confirm this Work has not been accepted for publication elsewhere, is not being considered for publication elsewhere and does not duplicate material already published. I confirm all authors consent to publication of this Work and authorise the granting of this licence.

For peer review only

An exploration of the impact of the Covid-19 pandemic on people with dementia and carers from Black and Minority Ethnic groups

Authors

Emily West [1*]
Pushpa Nair [2*]
Yolanda Barrado-Martin [2]
Kate Walters [2]
Nuriye Kupeli [1]
Elizabeth L Sampson [1,3]
Nathan Davies [2]

[1] Marie Curie Palliative Care Research Department, University College London, London, UK
[2] Department of Primary Care and Population Health, Royal Free Campus, University College London, London, UK
[3] Barnet Enfield, Haringey Mental Health Trust Liaison Psychiatry Team, North Middlesex University Hospital, London, UK

*Both authors contributed equally and share first authorship

Corresponding Author:

Emily West – emily.west@ucl.ac.uk

UCL Division of Psychiatry
6th Floor, Wing A, Maple House,
149 Tottenham Court Road,
London W1T 7NF

Funding

This work was supported by the Economic and Social Research Council [grant number: ES/V003720/1]. Nuriye Kupeli is supported by Alzheimer's Society Junior Fellowship grant funding (Grant Award number: 399 AS-JF-17b-016). Nathan Davies is supported by Alzheimer's Society Junior Fellowship grant funding (Grant Award number: 399 AS-JF-16b-012). Kirsten Moore and Elizabeth L Sampson are supported by Marie Curie core grant (number MCCC-FCO-16-U).

Keywords - Dementia, Carers, BAME, Qualitative, Interviews

Word Count - 5898

For peer review only

Abstract

Introduction

Despite community efforts to support and enable older and vulnerable people during the Covid-19 pandemic, many people with dementia and their family carers are still finding it difficult to adjust their daily living in light of the disruption that the pandemic has caused. There may be needs specific to BAME populations in these circumstances that remain thus far unexplored.

Objectives

The aim of the study was to explore the effects of the Covid-19 pandemic people living with dementia and their family carers from BAME backgrounds, in relation to their experiences of community dementia care, and the impact on their daily lives.

Design

15 participants (persons with dementia and carers) were recruited for semi-structured qualitative interviews. Respondents were from South Asian and Afro-Caribbean backgrounds. We used thematic analysis to analyze our data from a constructivist perspective, which emphasizes the importance of multiple perspectives, contexts and values.

Results

Eight key themes, with sub-themes, were identified– fear and anxiety, food and eating (encompassing food and shopping + eating patterns), isolation and identity, community and social relationships, adapting to covid-19, social isolation and support structures and medical interactions.

Discussion

This paper covers unique and underexplored topics in a COVID-19 vulnerable group. There is limited work with these groups in the UK, and this is especially true in COVID-19. The results showed that such impacts were far-reaching and affected not only day-to-day concerns but also care decisions with long-ranging consequences, and existential interests around fear, faith, death and identity.

Article Summary

Strengths and Limitations

- This paper covers underexplored topics at-risk groups during COVID-19. BAME groups are both particularly vulnerable and understudied in this context.

For peer review only

- However, there are certain limitations to our approach. This study focussed on South Asian and Afro-Caribbean groups, and views may not be generalisable to other minority groups.
- However, the spirit of the findings – i.e. ensuring cultural appropriateness in services and interventions – is widely applicable.
- We also had a likely self-selecting tech-savvy study population. Due to some aspects of recruitment (via online registries) and the mode of study (interview via Zoom or other video call) we reached people who already had a decent level of access to technology, and/or could be facilitated in this by an informal carer. This is not widely applicable to all older adults.

For peer review only

Background

Over 850,000 people in the UK have dementia and an estimated 25,000 of these come from Black, Asian and Minority Ethnic (BAME) groups, defined in the UK as non white British [1]. The National Audit Office[2] has proposed that special dementia services may be needed to support these groups. There is evidence that people from ethnic minority backgrounds seek less support from services [3]possibly as a result of increased stigma, alternative health beliefs, reduced knowledge about dementia and a perceived lack of culturally-tailored care[3–6]. There is also evidence that certain minority backgrounds may be less likely to receive a diagnosis of dementia, despite increased risk [7,8]. There is often reluctance amongst BAME communities to enter residential or nursing care, with most care being undertaken by family members in the community [9]. This may be due to cultural norms and beliefs about caring for older family members, and multigenerational living already being more common. Fears about culturally sensitive or appropriate care may also come into play. Indeed, the majority of people living with dementia live in the community in their own homes or with family [10]. In the UK, there are currently around 700,000 family members and friends acting as primary carers (i.e. providing the majority of care) [11].

Despite community efforts to support and enable older and vulnerable people during the Covid-19 pandemic, many people with dementia and their family carers are still finding it difficult to adjust their daily living in light of the disruption that the pandemic has caused [12]. There may be needs specific to BAME populations in these circumstances that remain thus far unexplored.

Aspects of dementia care, such as caring arrangements and access to health and social care have been widely affected. We wanted to explore the effects of the pandemic on general wellbeing, of both people with dementia and carers from BAME communities, particularly in light of raised concerns over higher incidence of Covid-19 in BAME groups and higher risks of complications [13]. BAME communities have also been found to be particularly COVID-19 vulnerable, with people from a Bangladeshi background being twice as likely to die from COVID-19 as those from a White British background. Similarly, people of a black Caribbean or other black background are at 10-50% increased risk of death compared to White British [14]. Older adults of a BAME background have also expressed that they are more likely to decline a COVID-19 vaccine than white Britons [15,16], which suggests that this gap may yet widen further. In light of these facts, this study concentrates on BAME community members of South Asian and Afro-Caribbean backgrounds, to help us understand what the real challenges are in order to develop ways to promote tailored support throughout the pandemic. This study focusses on community care, as this model of help-seeking was heavily promoted to caregivers as pressure on acute services increased.

The aim of the study was to explore the effects of the Covid-19 pandemic people living with dementia and their family carers from BAME backgrounds, in relation to their experiences of community dementia care, and the impact on their daily lives.

For peer review only - http://bmjopen.bmj.com/site/about/guidelines.xhtml

Research Designs and Methods

Participants

This study was a part of the ENDEMIC: dEmeNtia and DEcision MakIng during Covid19 programme, funded by the Economic and Social Research Council, aimed at developing an evidence-based decision tool to support family carers and people with dementia to make these difficult decisions during COVID-19. We recruited 15 participants in total, 11 family carers and four persons living with dementia. Participants were recruited from a variety of sources, including a previous studies, online dementia research websites (such as Join Dementia Research), social media, and interested relevant local/national carer organisations. Participants were recruited primarily from in and around Greater London, due to the high proportion of BAME communities in this area, although we did also recruit participants from further afield, in order to sample regions with different ethnicities, socioeconomic backgrounds, and variable access to healthcare and dementia services. The study was approved by UCL Research Ethics Committee (17623/002).

Inclusion Criteria

- Current carers from South Asian and Afro-Caribbean backgrounds providing unpaid care to someone living with dementia.
- People over the age of 65 with dementia from South Asian or Afro-Caribbean backgrounds, who have dementia but the capacity to consent

Exclusion Criteria

- Carers with cognitive impairment or bereaved carers
- People with dementia who have been diagnosed <6 months ago
- Participants who do not have mental capacity to provide informed consent
- Participants who were not from South Asian or Afro-Caribbean backgrounds were not included in the study.

Recruitment

Participants recruited by local/national dementia/carer networks were initially approached by the organisation (in line with their local organisation policies and guidelines), or directly by the research team. All interested participants were asked to contact the study team directly or gave permission to be sent a copy of the study advert, study information leaflet and consent form (according to participant's preferred method of communication – via email or post). Interested participants were encouraged to contact the research team via email or telephone and were then all telephone screened against the study criteria and given an opportunity to ask questions.

Consent forms were completed electronically and sent back to the research team via email, where possible. They were then countersigned by the research team and returned to the participant via email, to keep for their records. Special accessible consent forms were made available for people with dementia. If participants were unable to complete the consent

For peer review only

form electronically, then consent was taken verbally and audio-recorded prior to the start of the interview. A hard copy of the consent form was then filled in, signed by the researcher and emailed or posted back to the participant, to keep for their records.

Patient and Public Involvement

A patient and public involvement panel was consulted throughout the research process. The panel provided input on devising the research question, leading us to focus on community care and experience. The panel also gave feedback on the design of the study – namely managing the burden of interviews on persons with dementia and carers - formulation of the topic guide and interpretation of findings.

Data Collection

We collected data through individual semi-structured interviews, conducted remotely over telephone (n=13) or via secure video technologies (Microsoft Teams, n=2). Interviews were conducted by PN (n=14, an academic GP) and EW (n= 1, a researcher). Interviews lasted approximately one hour (range: 35 – 75 minutes) and were audio-recorded using a Dictaphone. The interview topic guide was developed with the input of the research team, including our patient and public involvement (PPI) representatives, and was modified as interviews progressed. The topic guide explored the impact of the Covid-19 pandemic on BAME carers and people with dementia, considering: daily routines, eating and drinking, wellbeing, access to dementia services, healthcare and social care, and exploring what extra support they would have found useful.

A distress protocol was devised in case participants became distressed during interviews, but we had no recourse to use this.

At the end of each interview, we collected demographic data on age, gender, marital status, ethnic group, country of birth, first language, education level and work. Audio-recorded interview data was transcribed verbatim, anonymized, and verified for accuracy by the interviewer.

Data analysis

We used thematic analysis to analyze our data from a constructivist perspective, which emphasizes the importance of multiple perspectives, contexts and values [17]. Our team included two academic GPs (PN, KW), a senior qualitative researcher (ND), and two early career researchers (EW, YB). PN developed a thematic framework, which was further refined with the input of YB, EW and ND. This framework was further refined by EW. EW then iteratively coded transcripts using NVivo 11 (ref) according to the thematic framework, and in discussion with the wider research team.

Results

Eight key themes, with sub-themes, were identified– fear and anxiety, food and eating (encompassing food and shopping + eating patterns), isolation and identity, community and

For peer review only

social relationships, adapting to covid-19, social isolation and support structures and medical interactions.

Fear and anxiety

PwD 01 It makes me worried, but at the same time, I accept that it is all over the world. Nobody will escape this corona. So, I accept it as God's will. I don't know what it is.

Fear and anxiety as a theme was discussed both explicitly and was reflected implicitly throughout other themes. This topic proved to be an overarching theme that was a foundation for other subjects discussed. Fear took many forms, from overarching existential fear surrounding the concept of risk or serious illness or death, to anxieties about specific situations or susceptibilities – such as risks inherent in using public transport, going to work or seeing family. Many fears were expressed in conversations about comorbidities – factors that made the carer or person with dementia more susceptible to contracting COVID-19 or less likely to respond well to treatment. There was some awareness in the group of respondents that BAME individuals were at generally higher risk due to being overrepresented in frontline jobs. This was sometimes attributed to having more limited work options available. This was an additional form of anxiety for many, as was the worry that they could bring the virus into contact with those with dementia by virtue of these roles.

PwD 02 Yes, I do, yes, because I think our people get the worst of all worlds. And you know, we may say, well, if you don't like the job get yourself another job. But where are you going to get it? And if you can get it, who is going to give it to you? This is it

Food and eating

One of the most discussed subjects amongst carers was food and eating, which was specifically explored in interviews. Many issues around food were discussed, and were split into two main themes – shopping for food, and eating food.

Food Shopping

Food shopping was a cause of some stress for many carers, specifically in early stages of the first lockdown where rules around priority access weren't fully in place. There was also considerable anxiety around the safety of shopping for food, with fears around exposing the person with dementia to COVID-19 through a lack of social distancing in supermarkets, or bringing the virus into the home on food. Many carers reported that they sanitised food bought in supermarkets to mitigate this risk. These anxieties made food shopping a time consuming and fraught process for carers. Similarly, a number of persons with dementia expressed frustration that they no longer had the independence to go to supermarkets and pick out what they wanted due to shielding.

Carer 10 But the queues, I had to go to [supermarket] … You don't get it so much now, but the queues were a mile long. You didn't know when you went shopping how long the queue was to get in. All of that.

For peer review only

Eating Patterns

Changes to food behaviours – defined widely as behaviours that encompass food shopping, preparation and mealtimes - brought on by the pandemic were also reflected in eating patterns. It was widely reported both by our respondents and in the media that the food parcels given out by councils to vulnerable members of the community (which included many of our sample) were not ethnically targeted or sometimes even appropriate. For example, respondents following specific diets – vegetarian, halal – received many items that they could not eat. A number of Caribbean respondents reported that culturally-common foods such as plantain and yams were not available to them, especially early in the pandemic.

Carer 09 Yes, I would have thought so. Basically, people of their own culture, like Indians, we would prefer to have obviously Indian food. That doesn't mean that we don't like Western food or Chinese food, for that matter. Of course. That is there. I wouldn't refuse chole puri [a chickpea dish] [any day.

Some respondents reported that they adapted their diet based on foods available or given to them and ate foods that they hadn't before. A number of persons with dementia also reported temporarily cutting unavailable foods out of their diets, rather than going to greater lengths to secure access to them.

PwD 04 Yes but also the tinned food. I never used to eat things like that and I used to eat the right food, fresh fruit and fresh veg and I love fish. Fish and stuff. And it's just like tins of tuna. Since the lockdown I've had two corned beef and it's not that bad when I eat it, but for my age, so I've got to watch what I'm doing but I had no choice

Some eating-focussed adaptations to the pandemic were positive, with carers and person with dementia reporting that diets had improved or become healthier in this time due to having more time to make fresh meals. Carers often felt reassured that they could better monitor the person with dementia's food intake and dietary habits. Being able to express care through cooking, share food and ensure nutritious food that was likely to be appealing to the person with dementia was seen as positive. However, the extra work and worry associated with being responsible for the person with dementia's food intake was often seen as a negative.

Carer 07 So it was a heavy impact on me and I think again it's that being pulled in two ways. Being worried about myself as an individual, but actually this is my job and I've got to do it before anybody says anything

Isolation and identity

All respondents interviewed talked about their own wellbeing and how this has changed over the course of the pandemic. Many carers reported feeling strained, without their usual outlets and opportunities to have an identity outside of being a carer. A loss of freedom was particularly reported among older spousal carers, as they also often had comorbidities that

For peer review only - http://bmjopen.bmj.com/site/about/guidelines.xhtml

put them at risk. This made some couples heavily dependent on outside or familial help for tasks that they had previously been able to manage independently. Reduced social interaction was widely reported by both groups of respondents, and often behind this was a fear of contracting COVID-19 and transmitting this to loved ones. This often led carers to report that they adhered to self-imposed lockdowns more strict than those imposed by the government.

Carer 04 Just that carers feel isolated, even before the lockdown. So, in the lockdown there's a lot of pressure to follow the guidelines, to do everything with the limitations. Things like going to the grocery [store], to buy groceries is extremely stressful because having to get everything so you don't have to keep going out all the time.

As with all themes, experiences of isolation and redefining identities were very individualistic, so down-sides of a situation for some proved to be up-sides for others. For some this was related to financial savings, or having the ability and support to access online services and activities. For example, a number of creative solutions to cancelled activities and restrictions were reported, from going for an evening drive to attending Zoom worship services or online exercise classes. However, for some this took the form of a relaxing of social pressures. One spousal carer reported that lockdown actually enabled her to rest as she could no longer go out and run errands. In many cases there was also more family help available, and greater financial support able to be accessed. This was largely related to individual or family circumstances than culture per se.

Carer 10 I've got into gardening even more. I did a course in horticulture last year, and that has helped. So, I've just gone out and bought loads of plants. It's amazing actually. A lot of people have said this, but just buying a whole lot of plants and actually learning how to care for these plants.

Community and Social Relationships

Religion and cultural celebrations were important for many respondents, and being unable to go to church, temple or similar was very difficult for some. Many holidays, observances and celebrations were missed, these were considered usually very communal experiences. In their place, personal prayer gained a heightened importance. Rites of passage were also affected by COVID-19, with weddings and baptisms being postponed and cancelled. Funerals were also widely affected, and this had particular significance in cultures where seeing the body, or burial within a certain timeframe is important. Similarly, having a large well-attended funeral is seen as a mark of respect in many communities, and it was upsetting to respondents that this was unable to happen during the pandemic when community elders died.

Carer 01 That's quite hard. In terms of traditionally, that's what we do. You have to say your goodbyes. And if you can't, that's the worst possible thing, really, if you can't go to somebody's funeral.

Social dynamics were discussed by many respondents. Many carers reported that being distanced from their children and extended family – particularly early in the pandemic

where tight restrictions on interactions had been applied during the first lockdown – contributed to their feelings of isolation and increased workload. Suddenly jobs which had previously been shared across a whole (extended) family now fell to one or two members, especially if some family members had high-risk jobs that put them in contact with many members of the public. Many older carers also reported that their children felt stressed about the risk of transmitting COVID-19 to older parents, and so reduced contact. This led to deeper feelings of social isolation.

Carer 06 Do you know what? We just prayed. Because we were being so safe, wearing gloves ourselves and putting slippers on. And me and my sister said to each other, if any of us became ill, we wouldn't go. The other one would carry the burden. So, we just prayed. We didn't think about it, actually. We just hoped it wouldn't happen

There were some positive adaptations to these issues, with people reporting that they had learned to use new devices or forms of technology such as Zoom, FaceTime and monitoring technologies for the first time. Some carers reported that they had encouraged or helped older relatives to use technology during the lockdown despite being unable to before, and that this had had a positive effect on their family and social dynamics.

Adapting to COVID-19

Both persons with dementia and carers were asked about their awareness of, and attitudes towards, COVID-19 during interviews. Most persons with dementia were reported by carers to have an awareness of their own higher risk due to COVID-19 susceptibility. Many also self-reported that they generally accepted the situation with a sense of things being outside of their control, "what will be will be":

PwD 03 I don't care about coronavirus because a lot depends on God.

When persons with dementia reported their own awareness of COVID-19 they either understood the situation well, or expressed an understanding of danger without the specificities of understanding the risk profile of an airborne pandemic virus. For example, there was generally a good understanding of the fact that usual life had shut down, shops were closed etc. but this was contrasted with continuing frustration over not being able to go out, see friends and partake in normal activities and routines.

Behavioural changes in persons with dementia – such as increasing depression, anxiety, aggression and sleeplessness - were reported by carers (who tended to be caring for people with more advanced dementia than the persons with dementia that we interviewed directly), and this was more often reported in persons with dementia who didn't have a good awareness of the reality of COVID-19. For these individuals, grasping aspects of changed rules and environments such as social distancing was difficult or impossible, and this in-turn was a source of stress for carers. Heightened anxiety was reported by carers in persons with dementia who both understood, and didn't understand, the full concept of the COVID-19 pandemic. Carers of persons with dementia who had the capacity to modify their own behaviour in relation to COVID-19 risk reported less of this type of anxiety. Carers of persons with dementia who did not practise such behaviour-modification ascribed

For peer review only

behavioural changes to a lack of routine and social interaction, and this was also associated with greater incidence of behaviours that challenge such as sleep/wake disturbance, aggression and depression.

Carer 04 Because often they will blame you for everything or be very aggressive. Especially when tensions are high and you're doing everything but still getting blamed and aggression directed towards you. And I think it's very easy for carers' mental health to decline very quickly in lockdown

Social Isolation and Support Structures

Different behaviours in accessing support structures were often related to burden. Many caregivers reported that they cancelled the services of paid carers or ancillary staff such as cleaners during the pandemic due to fear of exposing the person with dementia, which increased the sense of burden on partners and family members.

Carer 02 Coronavirus didn't bother me. Only for him. I stopped the carers for him. I stopped going for walks because of him, because he's at very high risk even now. But it didn't bother me. I said, if it has to come, it'll come. And if I have to die, I have to die. It didn't bother me at all. But its impact on life was changed, that we couldn't do things that we were doing.

In addition to this, almost all respondents reported that community dementia care services were largely absent during lockdown. This was acutely felt by both persons with dementia and carers. Before the pandemic, services like Dementia Hubs or day centres would provide structure to the week and allow respite time for carers. The absence of these services meant that carers struggled to find time for self-care and other activities outside of the person with dementia's capacity such as supermarket shopping. Persons with dementia also felt this loss, as their social interactions and activities were significantly lessened without them. Many of these services were reported to have started up again virtually as lockdown progressed, but many carers and persons with dementia reported finding it difficult to engage by this medium, and experienced a steep learning curve in setting it up. Those who did not have strong links with these types of services before the pandemic reported that they could not access them as a new service user during lockdown. A strong existing bond was seen to be key in accessing this particular form of help.

Carer 06 It's been terrible, because normally my mum would go on Dial-a-ride to the gurdwara, once a week. She'd go every Wednesday. And, obviously, the gurdwaras are closed. Because she used to go once a week and see all her friends, and that's been a big impact. And also, the day centres are all closed, and that has had an impact I think on her, because she's been quite isolated. I think she's been a bit lonely.

Access to support services in general were location-dependent. Some carers and persons with dementia reported having good access to supportive services such as carers, support lines and community support whereas others reported having none. Though many appreciated the community spirit that precipitated neighbours and local groups offering help, some were wary of the lack of regulation and safeguarding surrounding such services

delivered to vulnerable people. Discussion of differences in service provision often precipitated discussion of government handling of COVID-19. Though many were happy with communication and rules – and often phrased this in terms of appreciating that it was a difficult job in an uncertain situation – some were critical of the lack of clarity and provision provided by both central and local governments.

C04 But the government advice has been so ambiguous and changing. I feel like the lockdown didn't happen soon enough and I feel like the restrictions are being eased too quickly.

Medical Interactions

Many respondents had had interactions with medical services during the pandemic, whether as an inpatient or an outpatient. The majority of respondents had only outpatient medical interactions in this time. Most reported having good access to GP surgeries, though they did report missing face-to-face contact with their provider. In some cases, especially later in the pandemic, GPs were more flexible with in-person appointments and this was widely appreciated by respondents. Services allied to medicine - e.g. memory services, podiatry, hearing aid services, eye tests – were widely unavailable during the pandemic, and respondents reported falling behind on regularly scheduled care, and being anxious about this. This was also frequently reported regarding hospital appointments.

Carer 10 You can't just pick up and go to the GP and say, this is a problem or that. So, it felt like a bit of a fight, those everyday things. It just added to it.

There were a wide range of experiences related to hospital care. In the UK, hospital visiting has not been allowed during COVID-19. All respondents who had experienced the person with dementia requiring inpatient care reported that this was particularly disorienting for the person with dementia due to not being permitted to have family or other support present during admission. For carers, this was especially stressful when they felt that communication with the medical team was impaired. Some respondents felt "left-out" or unable to access information about the care or prognosis of the person with dementia. However, some reported exceptional engagement with hospital staff, and appreciative of the way that they facilitated the person with dementia to access communications technology during their admission.

Carer 09 It was quite traumatising for me as well, because I wasn't there next to her, and I just didn't know what the hell was going on. Waiting for the doctor to give me a call after they had visits and so on. It wasn't pleasant at all.

Planning and decision-making

Most carers reported that they hadn't had care-planning conversations with the person with dementia at the beginning of the pandemic, and that decisions (when required) were made in collaboration with the medical team and extended family. Being confronted with the need to make these decisions in a time of heightened risk and lessened service availability

For peer review only

was a key source of stress for many. Persons with dementia who were interviewed directly tended to express a similar “decisions will be made when they need to be” outlook.

Carer 08 *My only concern was that if anything happened to him, I didn't want him to go to the hospital. I was scared of this Corona*

Decisions were typically made with the significance of new regulations – e.g. on visiting, contact and triage – at the forefront of the decision-making process. This meant choosing to forgo certain regular treatments even when services opened back up, as well as choosing home care over hospital or care-home admittance or limiting the involvement of outside carers.

For peer review only

Discussion

This paper covers unique and underexplored topics in a COVID-19 vulnerable group. Focussed research on the needs of often-disadvantaged people in an emergency health situation are very much needed. There is limited work with these groups in the UK, and this is especially true in COVID-19. Themes were wide-ranging and identified a number of ways that the COVID-19 pandemic has impacted BAME persons with dementia and carers with regards to their experiences of dementia community care, and impact on their everyday lives. The results showed that such impacts were far-reaching and affected not only day-to-day concerns but also care decisions with long-ranging consequences, and existential interests around fear, faith, death and identity.

Support, or a lack thereof

BAME groups have been disproportionately affected by the COVID-19 pandemic in the UK [18]. In conjunction with this, past meta-analysis has identified significant barriers faced by members of BAME groups in accessing dementia care [5] both at the community and the service level. Many of our respondents, both persons with dementia and carers, spoke about their own experiences of help-seeking - whether in light of a lack of available services, or difficulty in the process of seeking them. Uncertainty around service provision, a lack of access without pre-pandemic relationships with services and practical difficulties in accessing support digitally were all sources of anxiety. Some patients and caregivers will not have these relationships as the pandemic has changed their needs, and thus required support levels. Independently of COVID-19, some people's needs will also naturally have changed due to decline over time.

Both persons with dementia and carers remarked on feeling great loss at a lack of routine and identity outside of the caring relationship. Past research [19] has shown strong correlation between carer burden and subsequent depression and wellbeing. This has been shown to be related to use of outside sources of support. In a circumstance where outside support has been found to be difficult to access across the board, particular attention should be paid to the burden of burnout and depression in carers moving forwards. Similarly, in persons with dementia independence and autonomy have been posited as key in maintaining positive identities in bound circumstances - namely care homes [20]. These will have been affected by COVID-19 on both micro and macro levels, from government regulations to changes to routines made on the family level in response to fear and anxiety.

A key form of support that was specifically probed in interviews, was behaviour around food and eating. Food insecurity throughout the pandemic has been widespread, and related to external factors such as employment security, stability of housing and isolation. Barker and Russell [21] identify a number of groups who were particularly at risk of food insecurity under the first COVID-19 lockdown, of which our respondents typically occupy at least two – namely being members of minority populations, and having or being a carer for somebody with a chronic illness. Thus, many respondents had been recipients of government-supplied food parcels, which were standardised and tailored towards a typically White British diet.

For peer review only

Food and eating behaviours are inextricably tied-up with ethnic and cultural identity, and the lack of availability of culturally appropriate food and ingredients during the first COVID-19 lockdown was the source of some distress for a number of interviewees.

This concern was also echoed in reference to community support and social interaction. Many reported a lack of being able to engage with usual forms of rites of passage, cultural milestones or celebrations with their community, and this in-turn added to feelings of isolation and lack of identity. Often, attendance at rites of passage – particularly funerals – signifies a person's social standing within the community. Thus the impact of having a sparsely-attended funeral is upsetting for those left behind and may contribute to compounded and complex grief [22]

These factors, in conjunction, constitute a form of double-disadvantaging whereby members of BAME communities living with or caring for those with dementia are not only disproportionately affected by the COVID-19 pandemic through systemic deprivation, but also by known barriers to service access, support or disenfranchisement through lack of access to culturally and ethnically appropriate provision, genetic factors and overrepresentation in frontline occupations.

Making plans and decisions

The majority of respondents, both persons with dementia and carers, had not made key medical or care decisions in advance of the COVID-19 pandemic. Previous research suggests that there are several reasons for such conversations not taking place in advance, from an expectation that family members will decide what is best [23], to a lack of tailored interventions that positively promote advance decision-making in minority groups [24]. Many carers reported feeling overwhelmed by information in the media about the level of risk, and potential lack of provision, for older people regarding medical care. A continued cause for concern was the restriction of visitors in acute medical and tertiary care settings. Several interviewees, both persons with dementia and carers, remarked that medical interactions had been especially stressful in light of a person with diminished capacity not having a family member to help them communicate and advocate in the medical setting. A COVID-19 prompted rapid-review of evidence around decision-making in older adults [25] identified the need for interventions to be culturally tailored, and that several studies highlighted a lack of positive response among BAME communities to generic planning interventions. Data from the interviews in this paper support these findings - from differing familial roles in decision-making to the need to recognise culturally specific ideas around autonomy and "what is proper".

Adapting to COVID-19

Adapting to new restrictions and ways of living under the first COVID-19 lockdown in the UK proved to be challenging for many, but those living with dementia and those involved in caring for persons with dementia faced extra challenges around care provision, help-seeking and lack of service availability. This in some cases is also compounded by difficulties in learning the skills required to access services remotely. Geibel et al [26]identified a significant reduction in support service usage amongst a population of persons with

For peer review only

dementia and their carers in the general population. This has potential significant future effects on distress and mental wellbeing levels in both persons with dementia and carers. Burden of behavioural and psychological symptoms of dementia (BPSD) have been consistently found to be associated with an increased risk of nursing home admission [27]. Thus, the increase in those dealing with BPSD in persons with dementia under lockdown may prove to have significant repercussions on public health through potential increase in future care home admissions.

Technology was used to access a huge range of services and interactions within our respondent group, from healthcare to social and religious ceremonies. As our respondents in a number of cases were recruited from an online dementia research registry, our sample may skew more tech-savvy than most. Many outreach groups strongly promoted digital literacy training during the pandemic, which may also have had a positive effect. However, those without the capacity to access digital services may end up further disadvantaged. Previous literature [28,29] has highlighted the importance of enabling older adults with dementia and carers to use technology to promote social interaction, and the unique circumstances of the COVID-19 pandemic may have hastened the need for widespread digital inclusion – both for social needs and wider functions such as maintaining contact with those in hospitals and care homes, attending telehealth appointments and ordering groceries.

Strengths and Limitations

This paper covers underexplored topics at-risk groups during COVID-19. BAME groups are both particularly vulnerable and understudied in this context. However, there are certain limitations to our approach. This study focussed on South Asian and Afro-Caribbean groups, and views may not be generalisable to other minority groups. However, the spirit of the findings – i.e. ensuring cultural appropriateness in services and interventions – is widely applicable. We also had a likely self-selecting tech-savvy study population. Due to some aspects of recruitment (via online registries) and the mode of study (interview via Zoom or other video call) we reached people who already had a decent level of access to technology, and/or could be facilitated in this by an informal carer. This is not widely applicable to all older adults.

Conclusions

BAME communities, including persons with dementia and their carers, in the UK have been heavily impacted by the COVID-19 pandemic. These impacts have been multi-faceted, beginning with susceptibility to the virus and encompassing aspects of life as diverse and service access, social relationships and access to culturally specific resources. Underpinning many of these experiences has been a paucity of support. There are potentially long-reaching effects of these experiences that will be uncovered as the pandemic continues and beyond, including increased support needs of patients and carers, looking at promoting equity of service access and provision and exploring the role of technology in health and social care in the future. This paucity of support should be addressed to prevent potential widening inequalities and negative impacts on health and wellbeing.

For peer review only

Conflicts of Interest

The authors named certify that they have NO affiliations with, or involvement in, any organization or entity with any financial or non-financial interest in the subject matter or materials discussed in this manuscript.

Author Statement

PN performed the majority of interviews and developed the coding framework. EW performed extra interviews, contributed to the coding framework, performed analysis and drafted the manuscript. YBM refined the coding framework and contributed to the manuscript. KW, NK and ES contributed to the manuscript. ND contributed to the manuscript and provided adjudication on analysis. Thanks to our patient and public involvement panel for providing valuable input into study design and strategy.

For peer review only

Bibliography

1. Alzheimer, Society. *Dementia UK Second Edition - Overview*., 2007.
2. Improving Dementia Services in England: An Interim Report - Great Britain. National Audit Office - Google Books.
3. Cooper C, Tandy AR, Balamurali TBS *et al.* A systematic review and meta-analysis of ethnic differences in use of dementia treatment, care, and research. *Am J Geriatr Psychiatry* 2010;**18**:193–203.
4. Daker-White G, Beattie AM, Gilliard J *et al.* Minority ethnic groups in dementia care: A review of service needs, service provision and models of good practice. *Aging Ment Heal* 2002;**6**:101–8.
5. Kenning C, Daker-White G, Blakemore A *et al.* Barriers and facilitators in accessing dementia care by ethnic minority groups: A meta-synthesis of qualitative studies. *BMC Psychiatry* 2017;**17**:1–13.
6. Mukadam N, Cooper C, Livingston G. A systematic review of ethnicity and pathways to care in dementia. *Int J Geriatr Psychiatry* 2011;**26**:12–20.
7. Pham TM, Petersen I, Walters K *et al.* Trends in dementia diagnosis rates in uk ethnic groups: Analysis of uk primary care data. *Clin Epidemiol* 2018;**10**:949–60.
8. Adelman S, Blanchard M, Livingston G. A systematic review of the prevalence and covariates of dementia or relative cognitive impairment in the older African-Caribbean population in Britain. *Int J Geriatr Psychiatry* 2009;**24**:657–65.
9. Giebel CM, Zubair M, Jolley D *et al.* South Asian older adults with memory impairment: improving assessment and access to dementia care. *Int J Geriatr Psychiatry* 2015;**30**:345–56.
10. *Homecare and Dementia Dementia and Homecare: Driving Quality and Innovation*., 2015.
11. Lewis F, Karlsberg Schaffer S, Sussex J *et al. The Trajectory of Dementia in the UK-Making a Difference*., 2014.
12. Suárez-González A, Livingston G, Low LF *et al.* Impact and Mortality of COVID-19 on People Living With Dementia: Cross-Country Report. *Inst Aging Publ* 2020.
13. Razaq A, Harrison D, Barr B *et al.* BAME Covid-19 Deaths - what do we know?: rapid data & evidence review. 2020.
14. *Disparities in the Risk and Outcomes of COVID-19*., 2020.
15. Bell S, Clarke R, Mounier-Jack S *et al.* Parents' and guardians' views on the acceptability of a future COVID-19 vaccine: A multi-methods study in England. *Vaccine* 2020;**38**:7789–98.
16. Paul E, Steptoe A, Fancourt D. Attitudes towards vaccines and intention to vaccinate against COVID-19: Implications for public health communications. *Lancet Reg Heal - Eur* 2020:100012.
17. The Constructivist Credo - Yvonna S Lincoln, Egon G Guba - Google Books.
18. Rose T, Mason K, Pennington A *et al.* Inequalities in COVID19 mortality related to ethnicity and socioeconomic deprivation. *medRxiv Prepr Serv Heal Sci* 2020:2020.04.25.20079491.
19. Miller VJ, Killian MO, Fields N. Caregiver identity theory and predictors of burden and depression: Findings from the REACH II study. *Aging Ment Health* 2020;**24**:212–20.
20. Paddock K, Brown Wilson C, Walshe C *et al.* Care Home Life and Identity: A Qualitative Case Study. *Gerontologist* 2019;**59**:655–64.
21. Barker M, Russell J. Feeding the food insecure in Britain: learning from the 2020 COVID-

For peer review only

19 crisis. *Food Secur* 2020;**12**:865–70.
22. Kokou-Kpolou CK, Fernández-Alcántara M, Cénat JM. Prolonged Grief Related to COVID-19 Deaths: Do We Have to Fear a Steep Rise in Traumatic and Disenfranchised Griefs? *Psychol Trauma Theory, Res Pract Policy* 2020;**12**:S94.
23. De Souza J, Gillett K, Froggatt K *et al.* Perspectives of elders and their adult children of Black and minority ethnic heritage on end-of-life conversations: A meta-ethnography. *Palliat Med* 2020;**34**:195–208.
24. Kwak J, Haley WE. Current Research Findings on End-of-Life Decision Making Among Racially or Ethnically Diverse Groups. *Gerontologist* 2005;**45**:634–41.
25. West E, Moore K, Kupeli N *et al.* Rapid review of decision-making for place of care and death in older people: lessons for COVID-19. *Age Ageing* 2020:1–13.
26. Giebel C, Cannon J, Hanna K *et al.* Impact of COVID-19 related social support service closures on people with dementia and unpaid carers: a qualitative study. *Aging Ment Health* 2020:1–8.
27. Toot S, Swinson T, Devine M *et al.* Causes of nursing home placement for older people with dementia: A systematic review and meta-analysis. *Int Psychogeriatrics* 2017;**29**:195–208.
28. Davies N, Iliffe S, Hopwood J *et al.* The key aspects of online support that older family carers of people with dementia want at the end of life: A qualitative study. *Aging Ment Heal* 2020;**24**:1654–61.
29. Holthe T, Halvorsrud L, Karterud D *et al.* Usability and acceptability of technology for community-dwelling older adults with mild cognitive impairment and dementia: A systematic literature review. *Clin Interv Aging* 2018;**13**:863–86.

For peer review only

BMJ Open

An exploration of the impact of the Covid-19 pandemic on people with dementia and carers from Black and Minority Ethnic groups

Journal:	*BMJ Open*
Manuscript ID	bmjopen-2021-050066.R1
Article Type:	Original research
Date Submitted by the Author:	31-Mar-2021
Complete List of Authors:	West, Emily; University College London,<br>Nair, Pushpa; University College London, Department of Primary Care and Population Health<br>Barrado-Martin, Yolanda; University College London, Department of Primary Care and Population Health<br>Walters, Kate; University College London, Primary Care and Population Health<br>Kupeli, Nuriye; Marie Curie Palliative Care Research Unit, UCL Division of Psychiatry<br>Sampson, Elizabeth; University College London, Division of Psychiatry<br>Davies, Nathan; UCL, Department of Primary Care and Population Health
**Primary Subject Heading**:	Public health
Secondary Subject Heading:	Health services research, Palliative care, Patient-centred medicine
Keywords:	Dementia < NEUROLOGY, Adult palliative care < PALLIATIVE CARE, PALLIATIVE CARE

SCHOLARONE™
Manuscripts

I, the Submitting Author has the right to grant and does grant on behalf of all authors of the Work (as defined in the below author licence), an exclusive licence and/or a non-exclusive licence for contributions from authors who are: i) UK Crown employees; ii) where BMJ has agreed a CC-BY licence shall apply, and/or iii) in accordance with the terms applicable for US Federal Government officers or employees acting as part of their official duties; on a worldwide, perpetual, irrevocable, royalty-free basis to BMJ Publishing Group Ltd ("BMJ") its licensees and where the relevant Journal is co-owned by BMJ to the co-owners of the Journal, to publish the Work in this journal and any other BMJ products and to exploit all rights, as set out in our licence.

The Submitting Author accepts and understands that any supply made under these terms is made by BMJ to the Submitting Author unless you are acting as an employee on behalf of your employer or a postgraduate student of an affiliated institution which is paying any applicable article publishing charge ("APC") for Open Access articles. Where the Submitting Author wishes to make the Work available on an Open Access basis (and intends to pay the relevant APC), the terms of reuse of such Open Access shall be governed by a Creative Commons licence – details of these licences and which Creative Commons licence will apply to this Work are set out in our licence referred to above.

Other than as permitted in any relevant BMJ Author's Self Archiving Policies, I confirm this Work has not been accepted for publication elsewhere, is not being considered for publication elsewhere and does not duplicate material already published. I confirm all authors consent to publication of this Work and authorise the granting of this licence.

For peer review only

An exploration of the impact of the COVID-19 pandemic on people with dementia and carers from Black and Minority Ethnic groups

Authors

Emily West [1*]
Pushpa Nair [2*]
Yolanda Barrado-Martin [2]
Kate Walters [2]
Nuriye Kupeli [1]
Elizabeth L Sampson [1,3]
Nathan Davies [2]

[1] Marie Curie Palliative Care Research Department, University College London, London, UK
[2] Department of Primary Care and Population Health, Royal Free Campus, University College London, London, UK
[3] Barnet Enfield, Haringey Mental Health Trust Liaison Psychiatry Team, North Middlesex University Hospital, London, UK

*Both authors contributed equally and share first authorship

Corresponding Author:

Emily West – emily.west@ucl.ac.uk

UCL Division of Psychiatry
6th Floor, Wing A, Maple House,
149 Tottenham Court Road,
London W1T 7NF

For peer review only

Funding

This work was supported by the Economic and Social Research Council [grant number: ES/V003720/1]. Nuriye Kupeli is supported by Alzheimer's Society Junior Fellowship grant funding (Grant Award number: 399 AS-JF-17b-016). Nathan Davies is supported by Alzheimer's Society Junior Fellowship grant funding (Grant Award number: 399 AS-JF-16b-012). Kirsten Moore and Elizabeth L Sampson are supported by Marie Curie core grant (number MCCC-FCO-16-U).

Keywords - Dementia, Carers, BAME, Qualitative, Interviews

Word Count - 5898

For peer review only - http://bmjopen.bmj.com/site/about/guidelines.xhtml

Abstract

Introduction

Despite community efforts to support and enable older and vulnerable people during the COVID-19 pandemic, many people with dementia and their family carers are still finding it difficult to adjust their daily living in light of the disruption that the pandemic has caused. There may be needs specific to Black, Asian and minority ethnic (BAME) populations in these circumstances that remain thus far unexplored.

Objectives

The aim of the study was to explore the effects of the COVID-19 pandemic people living with dementia and their family carers from BAME backgrounds, in relation to their experiences of community dementia care, and the impact on their daily lives.

Design

15 participants (persons with dementia and carers) were recruited for semi-structured qualitative interviews. Respondents were from South Asian and Afro-Caribbean backgrounds. We used thematic analysis to analyze our data from a constructivist perspective, which emphasizes the importance of multiple perspectives, contexts and values.

Results

There was a number of ways that the COVID-19 pandemic has impacted BAME persons with dementia and carers with regards to their experiences of dementia community care, and impact on their everyday lives. In particular we identified eight key themes, with sub-themes: – fear and anxiety, food and eating (encompassing food and shopping and eating patterns), isolation and identity, community and social relationships, adapting to **COVID**-19, social isolation and support structures and medical interactions. Fear and anxiety formed an overarching theme that encompassed all others.

Discussion

This paper covers unique and underexplored topics in a COVID-19 vulnerable group. There is limited work with these groups in the UK, and this is especially true in COVID-19. The results showed that such impacts were far-reaching and affected not only day-to-day concerns but also care decisions with long-ranging consequences, and existential interests around fear, faith, death and identity.

Article Summary

Strengths and Limitations

For peer review only - http://bmjopen.bmj.com/site/about/guidelines.xhtml

- This paper covers underexplored topics at-risk groups during COVID-19 -BAME groups are both particularly vulnerable and understudied in this context.
- Interviews were undertaken using Microsoft Teams, and online videoconferencing platform
- This study focussed on South Asian and Afro-Caribbean groups, and views may not be generalisable to other minority groups.
- Data were analysed using an iterative constructivist approach and coded thematically
- Analysis was performed by a diverse team

For peer review only

For peer review only - http://bmjopen.bmj.com/site/about/guidelines.xhtml

Background

Over 850,000 people in the UK have dementia and an estimated 25,000 of these come from Black, Asian and Minority Ethnic (BAME) groups, defined in the UK as non white British [1]. The National Audit Office[2] has proposed that special dementia services may be needed to support these groups. There is evidence that people from ethnic minority backgrounds seek less support from services [3]possibly as a result of increased stigma, alternative health beliefs, reduced knowledge about dementia and a perceived lack of culturally-tailored care[3–6]. There is also evidence that certain minority backgrounds may be less likely to receive a diagnosis of dementia, despite increased risk [7,8]. There is often reluctance amongst BAME communities to enter residential or nursing care, with most care being undertaken by family members in the community [9]. This may be due to cultural norms and beliefs about caring for older family members, and multigenerational living already being more common. Fears about culturally sensitive or appropriate care may also come into play. Indeed, the majority of people living with dementia live in the community in their own homes or with family [10]. In the UK, there are currently around 700,000 family members and friends acting as primary carers (i.e. providing the majority of care) [11].

Despite community efforts to support and enable older and vulnerable people during the COVID-19 pandemic, many people with dementia and their family carers are still finding it difficult to adjust their daily living in light of the disruption that the pandemic has caused [12]. There may be needs specific to BAME populations in these circumstances that remain thus far unexplored.

Aspects of dementia care, such as caring arrangements and access to health and social care have been widely affected. We wanted to explore the effects of the pandemic on general wellbeing, of both people with dementia and carers from BAME communities, particularly in light of raised concerns over higher incidence of COVID-19 in BAME groups and higher risks of complications [13]. BAME communities have also been found to be particularly COVID-19 vulnerable, with people from a Bangladeshi background being twice as likely to die from COVID-19 as those from a White British background. Similarly, people of a black Caribbean or other black background are at 10-50% increased risk of death compared to White British [14]. Older adults of a BAME background have also expressed that they are more likely to decline a COVID-19 vaccine than white Britons [15,16], which suggests that this gap may yet widen further. In light of these facts, this study concentrates on BAME community members of South Asian and Afro-Caribbean backgrounds, to help us understand what the real challenges are in order to develop ways to promote tailored support throughout the pandemic. This study focusses on community care, as this model of help-seeking was heavily promoted to caregivers as pressure on acute services increased.

The aim of the study was to explore the effects of the COVID-19 pandemic on people living with dementia and their family carers from BAME backgrounds, in relation to their experiences of community dementia care, and the impact on their daily lives.

For peer review only - http://bmjopen.bmj.com/site/about/guidelines.xhtml

Research Designs and Methods

Participants

This study was a part of the ENDEMIC: dEmeNtia and DEcision MakIng during COVID-19 programme, funded by the Economic and Social Research Council, aimed at developing an evidence-based decision tool to support family carers and people with dementia to make these difficult decisions during COVID-19. We recruited 15 participants in total, 11 family carers and four persons living with dementia. Participants were recruited from a variety of sources, including a previous studies, online dementia research websites (such as Join Dementia Research), social media, and interested relevant local/national carer organisations. Participants were recruited from across the UK, though primarily from in and around Greater London, due to the high proportion of BAME communities in this area.

Ethics Approval Statement

The study was approved by UCL Research Ethics Committee (17623/002).

Data Availability Statement

Data is available from the authors on request.

Inclusion Criteria

Current carers from South Asian and Afro-Caribbean backgrounds providing unpaid care to someone living with dementia, or people over the age of 65 with dementia from South Asian or Afro-Caribbean backgrounds, who have dementia but the capacity to consent.

Exclusion Criteria

Carers with cognitive impairment or bereaved carers. People with dementia diagnosed under 6 months from interview date. Participants who do not have mental capacity to provide informed consent. Participants who were not from South Asian or Afro-Caribbean backgrounds.

Recruitment

Participants recruited by local/national dementia/carer networks were initially approached by the organisation - this was done in line with their local organisation policies and guidelines and included email, newsletters and direct contact. Participants were also approached directly by the research team. These participants were identified through past involvement in studies and ongoing permission to contact, and contact was initially through email or telephone. ll interested participants were asked to contact the study team directly or gave permission to be sent a copy of the study advert, study information leaflet and consent form (according to participant's preferred method of communication – via email or post). Interested participants were encouraged to contact the research team via email or

For peer review only - http://bmjopen.bmj.com/site/about/guidelines.xhtml

telephone and were then all telephone screened against the study criteria and given an opportunity to ask questions.

Consent forms were completed electronically and sent back to the research team via email, where possible. They were then countersigned by the research team and returned to the participant via email, to keep for their records. Special accessible consent forms were made available for people with dementia. If participants were unable to complete the consent form electronically, then consent was taken verbally and audio-recorded prior to the start of the interview. A hard copy of the consent form was then filled in, signed by the researcher and emailed or posted back to the participant, to keep for their records.

Patient and Public Involvement

A patient and public involvement panel was consulted throughout the research process. The panel provided input on devising the research question, leading us to focus on community care and experience. The panel also gave feedback on the design of the study – namely managing the burden of interviews on persons with dementia and carers - formulation of the topic guide and interpretation of findings.

Data Collection

We collected data through individual semi-structured interviews, conducted remotely over telephone (n=13) or via secure video technologies (Microsoft Teams, n=2). Interviews were conducted by PN (F) (n=14, an academic GP) and EW (F) (n= 1, a researcher). Interviews lasted approximately one hour (range: 35 – 75 minutes) and were audio-recorded using a Dictaphone. The interview topic guide was developed with the input of the research team, including our patient and public involvement (PPI) representatives, and was modified as interviews progressed. The topic guide (Appendix 1) explored the impact of the COVID-19 pandemic on BAME carers and people with dementia, considering: daily routines, eating and drinking, wellbeing, access to dementia services, healthcare and social care, and exploring what extra support they would have found useful.

A distress protocol was devised in case participants became distressed during interviews, but we had no recourse to use this. All participants were able to converse in English, though proficiency levels varied. Both interviewers have experience in working internationally and with persons with communication difficulties. No extra formal interventions were required to facilitate interviews.

At the end of each interview, we collected demographic data on age, gender, marital status, ethnic group, country of birth, first language, education level and work. Audio-recorded interview data was transcribed verbatim, anonymized, and verified for accuracy by the interviewer.

Data analysis

We used inductive thematic analysis to analyze our data from a constructivist perspective, which emphasizes the importance of multiple perspectives, contexts and values [17]. Our

For peer review only - http://bmjopen.bmj.com/site/about/guidelines.xhtml

team included two academic GPs (PN, KW), a senior qualitative researcher (ND), and two early career researchers (EW, YB). PN developed a thematic framework, which was further refined with the input of YB, EW and ND. This framework was further refined by EW. EW then iteratively coded transcripts using NVivo 11 according to the thematic framework, and in discussion with the wider research team.

**Demographics**	**PwD N=4 (%)**	**Carers N=11 (%)**
Age		
Under 50	0	9
50-59	0	36
60-69	25	9
70-79	50	27
80+	25	18
Gender		
Male	50	9
Female	50	91
Marital Status		
Single	25	27
Married	25	64
Divorced	25	0
Widowed	25	9
Ethnic Group		
Caribbean	50	45
Indian	50	45
Pakistani	0	9
Country of Birth		
India	25	27
Jamaica	50	0
Barbados	0	9
Kenya	0	9
Trinidad	0	9
Uganda	25	9
UK		36
First Language		
English	50	45
Indian Languages (Bengali, Gujarati, Punjabi, Urdu)	50	54
Age left Education		
Age 17-20	0	36
Over 20	100	64

Table 1: Demographic characteristics of participants

Results

For peer review only - http://bmjopen.bmj.com/site/about/guidelines.xhtml

For peer review only

Demographic characteristics of participants are detailed in Table 1. In interviews, eight key themes, with sub-themes, were identified– fear and anxiety, food and eating (encompassing food and shopping + eating patterns), isolation and identity, community and social relationships, adapting to COVID-19, social isolation and support structures and medical interactions.

Fear and anxiety

PwD 01 (M,88) It makes me worried, but at the same time, I accept that it is all over the world. Nobody will escape this corona. So, I accept it as God's will. I don't know what it is.

Fear and anxiety as a theme was discussed both explicitly and was reflected implicitly throughout other themes. This topic proved to be an overarching theme that was a foundation for other subjects discussed. Fear took many forms, from overarching existential fear surrounding the concept of risk or serious illness or death, to anxieties about specific situations or susceptibilities – such as risks inherent in using public transport, going to work or seeing family. Many fears were expressed in conversations about comorbidities – factors that made the carer or person with dementia more susceptible to contracting COVID-19 or less likely to respond well to treatment. There was some awareness in the group of respondents that BAME individuals were at generally higher risk due to being overrepresented in frontline jobs. This was sometimes attributed to having more limited work options available. This was an additional form of anxiety for many, as was the worry that they could bring the virus into contact with those with dementia by virtue of these roles.

PwD 02 (F,75) Yes, I do, yes, because I think our people get the worst of all worlds. And you know, we may say, well, if you don't like the job get yourself another job. But where are you going to get it? And if you can get it, who is going to give it to you? This is it

Food and eating

One of the most discussed subjects amongst carers was food and eating, which was specifically explored in interviews. Many issues around food were discussed, and were split into two main themes – shopping for food, and eating food.

Food Shopping

Food shopping was a cause of some stress for many carers, specifically in early stages of the first lockdown where rules around priority access weren't fully in place. There was also considerable anxiety around the safety of shopping for food, with fears around exposing the person with dementia to COVID-19 through a lack of social distancing in supermarkets, or bringing the virus into the home on food. Many carers reported that they sanitised food bought in supermarkets to mitigate this risk. These anxieties made food shopping a time consuming and fraught process for carers. Similarly, a number of persons with dementia expressed frustration that they no longer had the independence to go to supermarkets and pick out what they wanted due to shielding.

For peer review only - http://bmjopen.bmj.com/site/about/guidelines.xhtml

Carer 10 (F,57)But the queues, I had to go to [supermarket] … You don't get it so much now, but the queues were a mile long. You didn't know when you went shopping how long the queue was to get in. All of that.

Eating Patterns

Changes to food behaviours – defined widely as behaviours that encompass food shopping, preparation and mealtimes - brought on by the pandemic were also reflected in eating patterns. It was widely reported both by our respondents and in the media that the food parcels given out by councils to vulnerable members of the community (which included many of our sample) were not ethnically targeted or sometimes even appropriate. For example, respondents following specific diets – vegetarian, halal – received many items that they could not eat. A number of Caribbean respondents reported that culturally-common foods such as plantain and yams were not available to them, especially early in the pandemic.

Carer 09 (F, 72) Yes, I would have thought so. Basically, people of their own culture, like Indians, we would prefer to have obviously Indian food. That doesn't mean that we don't like Western food or Chinese food, for that matter. Of course. That is there. I wouldn't refuse chole puri [a chickpea dish] [any day.

Some respondents reported that they adapted their diet based on foods available or given to them and ate foods that they hadn't before. A number of persons with dementia also reported temporarily cutting unavailable foods out of their diets, rather than going to greater lengths to secure access to them.

PwD 04 (F, 66) Yes but also the tinned food. I never used to eat things like that and I used to eat the right food, fresh fruit and fresh veg and I love fish. Fish and stuff. And it's just like tins of tuna. Since the lockdown I've had two corned beef and it's not that bad when I eat it, but for my age, so I've got to watch what I'm doing but I had no choice

Some eating-focussed adaptations to the pandemic were positive, with carers and person with dementia reporting that diets had improved or become healthier in this time due to having more time to make fresh meals. Carers often felt reassured that they could better monitor the person with dementia's food intake and dietary habits. Being able to express care through cooking, share food and ensure nutritious food that was likely to be appealing to the person with dementia was seen as positive. However, the extra work and worry associated with being responsible for the person with dementia's food intake was often seen as a negative.

Carer 07 (F, 57) So it was a heavy impact on me and I think again it's that being pulled in two ways. Being worried about myself as an individual, but actually this is my job and I've got to do it before anybody says anything

Isolation and identity

For peer review only - http://bmjopen.bmj.com/site/about/guidelines.xhtml

All respondents interviewed talked about their own wellbeing and how this has changed over the course of the pandemic. Many carers reported feeling strained, without their usual outlets and opportunities to have an identity outside of being a carer. A loss of freedom was particularly reported among older spousal carers, as they also often had comorbidities that put them at risk. This made some couples heavily dependent on outside or familial help for tasks that they had previously been able to manage independently. Reduced social interaction was widely reported by both groups of respondents, and often behind this was a fear of contracting COVID-19 and transmitting this to loved ones. This often led carers to report that they adhered to self-imposed lockdowns more strict than those imposed by the government.

Carer 04 (F, 29) Just that carers feel isolated, even before the lockdown. So, in the lockdown there's a lot of pressure to follow the guidelines, to do everything with the limitations. Things like going to the grocery [store], to buy groceries is extremely stressful because having to get everything so you don't have to keep going out all the time.

As with all themes, experiences of isolation and redefining identities were very individualistic, so down-sides of a situation for some proved to be up-sides for others. For some this was related to financial savings, or having the ability and support to access online services and activities. For example, a number of creative solutions to cancelled activities and restrictions were reported, from going for an evening drive to attending Zoom worship services or online exercise classes. However, for some this took the form of a relaxing of social pressures. One spousal carer reported that lockdown actually enabled her to rest as she could no longer go out and run errands. In many cases there was also more family help available, and greater financial support able to be accessed. This was largely related to individual or family circumstances than culture per se.

Carer 10 (F, 57) I've got into gardening even more. I did a course in horticulture last year, and that has helped. So, I've just gone out and bought loads of plants. It's amazing actually. A lot of people have said this, but just buying a whole lot of plants and actually learning how to care for these plants.

Community and Social Relationships

Religion and cultural celebrations were important for many respondents, and being unable to go to church, temple or similar was very difficult for some. Many holidays, observances and celebrations were missed, these were considered usually very communal experiences. In their place, personal prayer gained a heightened importance. Rites of passage were also affected by COVID-19, with weddings and baptisms being postponed and cancelled. Funerals were also widely affected, and this had particular significance in cultures where seeing the body, or burial within a certain timeframe is important. Similarly, having a large well-attended funeral is seen as a mark of respect in many communities, and it was upsetting to respondents that this was unable to happen during the pandemic when community elders died.

For peer review only - http://bmjopen.bmj.com/site/about/guidelines.xhtml

Carer 01 (F, 62) That's quite hard. In terms of traditionally, that's what we do. You have to say your goodbyes. And if you can't, that's the worst possible thing, really, if you can't go to somebody's funeral.

Social dynamics were discussed by many respondents. Many carers reported that being distanced from their children and extended family – particularly early in the pandemic where tight restrictions on interactions had been applied during the first lockdown – contributed to their feelings of isolation and increased workload. Suddenly jobs which had previously been shared across a whole (extended) family now fell to one or two members, especially if some family members had high-risk jobs that put them in contact with many members of the public. Many older carers also reported that their children felt stressed about the risk of transmitting COVID-19 to older parents, and so reduced contact. This led to deeper feelings of social isolation.

Carer 06 (F, 57) Do you know what? We just prayed. Because we were being so safe, wearing gloves ourselves and putting slippers on. And me and my sister said to each other, if any of us became ill, we wouldn't go. The other one would carry the burden. So, we just prayed. We didn't think about it, actually. We just hoped it wouldn't happen

There were some positive adaptations to these issues, with people reporting that they had learned to use new devices or forms of technology such as Zoom, FaceTime and monitoring technologies for the first time. Some carers reported that they had encouraged or helped older relatives to use technology during the lockdown despite being unable to before, and that this had had a positive effect on their family and social dynamics.

Adapting to COVID-19

Both persons with dementia and carers were asked about their awareness of, and attitudes towards, COVID-19 during interviews. Most persons with dementia were reported by carers to have an awareness of their own higher risk due to COVID-19 susceptibility. Many also self-reported that they generally accepted the situation with a sense of things being outside of their control, "what will be will be":

PwD 03 (M, 76) I don't care about coronavirus because a lot depends on God.

When persons with dementia reported their own awareness of COVID-19 they either understood the situation well, or expressed an understanding of danger without the specificities of understanding the risk profile of an airborne pandemic virus. For example, there was generally a good understanding of the fact that usual life had shut down, shops were closed etc. but this was contrasted with continuing frustration over not being able to go out, see friends and partake in normal activities and routines.

PwD 02 (F, 75) Because I've accepted it is something we cannot do anything about, and it's just not me. It's everybody now, everybody. That's the first time, things like that, where we are... The equality is really meant what it really says. We're all equal now. So, I don't worry too much because it's not me alone, it's everybody.

For peer review only - http://bmjopen.bmj.com/site/about/guidelines.xhtml

Behavioural changes in persons with dementia – such as increasing depression, anxiety, aggression and sleeplessness - were reported by carers (who tended to be caring for people with more advanced dementia than the persons with dementia that we interviewed directly), and this was more often reported in persons with dementia who didn't have a good awareness of the reality of COVID-19. For these individuals, grasping aspects of changed rules and environments such as social distancing was difficult or impossible, and this in-turn was a source of stress for carers. Heightened anxiety was reported by carers in persons with dementia who both understood, and didn't understand, the full concept of the COVID-19 pandemic. Carers of persons with dementia who had the capacity to modify their own behaviour in relation to COVID-19 risk reported less of this type of anxiety. Carers of persons with dementia who did not practise such behaviour-modification ascribed behavioural changes to a lack of routine and social interaction, and this was also associated with greater incidence of behaviours that challenge such as sleep/wake disturbance, aggression and depression.

Carer 04 (F, 29) Because often they will blame you for everything or be very aggressive. Especially when tensions are high and you're doing everything but still getting blamed and aggression directed towards you. And I think it's very easy for carers' mental health to decline very quickly in lockdown

Social Isolation and Support Structures

Different behaviours in accessing support structures were often related to burden. Many caregivers reported that they cancelled the services of paid carers or ancillary staff such as cleaners during the pandemic due to fear of exposing the person with dementia, which increased the sense of burden on partners and family members.

Carer 02 (F, 71) Coronavirus didn't bother me. Only for him. I stopped the carers for him. I stopped going for walks because of him, because he's at very high risk even now. But it didn't bother me. I said, if it has to come, it'll come. And if I have to die, I have to die. It didn't bother me at all. But its impact on life was changed, that we couldn't do things that we were doing.

In addition to this, almost all respondents reported that community dementia care services were largely absent during lockdown. This was acutely felt by both persons with dementia and carers. Before the pandemic, services like Dementia Hubs or day centres would provide structure to the week and allow respite time for carers. The absence of these services meant that carers struggled to find time for self-care and other activities outside of the person with dementia's capacity such as supermarket shopping. Persons with dementia also felt this loss, as their social interactions and activities were significantly lessened without them. Many of these services were reported to have started up again virtually as lockdown progressed, but many carers and persons with dementia reported finding it difficult to engage by this medium, and experienced a steep learning curve in setting it up. Those who did not have strong links with these types of services before the pandemic reported that they could not access them as a new service user during lockdown. A strong existing bond was seen to be key in accessing this particular form of help.

For peer review only - http://bmjopen.bmj.com/site/about/guidelines.xhtml

Carer 06 (F, 57) It's been terrible, because normally my mum would go on Dial-a-ride to the gurdwara, once a week. She'd go every Wednesday. And, obviously, the gurdwaras are closed. Because she used to go once a week and see all her friends, and that's been a big impact. And also, the day centres are all closed, and that has had an impact I think on her, because she's been quite isolated. I think she's been a bit lonely.

Access to support services in general were location-dependent. Some carers and persons with dementia reported having good access to supportive services such as carers, support lines and community support whereas others reported having none. Though many appreciated the community spirit that precipitated neighbours and local groups offering help, some were wary of the lack of regulation and safeguarding surrounding such services delivered to vulnerable people. Discussion of differences in service provision often precipitated discussion of government handling of COVID-19. Though many were happy with communication and rules – and often phrased this in terms of appreciating that it was a difficult job in an uncertain situation – some were critical of the lack of clarity and provision provided by both central and local governments.

C04 (F, 29) But the government advice has been so ambiguous and changing. I feel like the lockdown didn't happen soon enough and I feel like the restrictions are being eased too quickly.

Medical Interactions

Many respondents had had interactions with medical services during the pandemic, whether as an inpatient or an outpatient. The majority of respondents had only outpatient medical interactions in this time. Most reported having good access to GP surgeries, though they did report missing face-to-face contact with their provider. In some cases, especially later in the pandemic, GPs were more flexible with in-person appointments and this was widely appreciated by respondents. Services allied to medicine - e.g. memory services, podiatry, hearing aid services, eye tests – were widely unavailable during the pandemic, and respondents reported falling behind on regularly scheduled care, and being anxious about this. This was also frequently reported regarding hospital appointments.

Carer 10 (F, 57) You can't just pick up and go to the GP and say, this is a problem or that. So, it felt like a bit of a fight, those everyday things. It just added to it.

There were a wide range of experiences related to hospital care. In the UK, hospital visiting has not been allowed during COVID-19. All respondents who had experienced the person with dementia requiring inpatient care reported that this was particularly disorienting for the person with dementia due to not being permitted to have family or other support present during admission. For carers, this was especially stressful when they felt that communication with the medical team was impaired. Some respondents felt "left-out" or unable to access information about the care or prognosis of the person with dementia. However, some reported exceptional engagement with hospital staff, and appreciative of the way that they facilitated the person with dementia to access communications technology during their admission.

For peer review only - http://bmjopen.bmj.com/site/about/guidelines.xhtml

Carer 09 (M, 72) It was quite traumatising for me as well, because I wasn't there next to her, and I just didn't know what the hell was going on. Waiting for the doctor to give me a call after they had visits and so on. It wasn't pleasant at all.

Planning and decision-making

Most carers reported that they hadn't had care-planning conversations with the person with dementia at the beginning of the pandemic, and that decisions (when required) were made in collaboration with the medical team and extended family. Being confronted with the need to make these decisions in a time of heightened risk and lessened service availability was a key source of stress for many. Persons with dementia who were interviewed directly tended to express a similar "decisions will be made when they need to be" outlook.

Carer 08 (F, 85) My only concern was that if anything happened to him, I didn't want him to go to the hospital. I was scared of this Corona

Decisions were typically made with the significance of new regulations – e.g. on visiting, contact and triage – at the forefront of the decision-making process. This meant choosing to forgo certain regular treatments even when services opened back up, as well as choosing home care over hospital or care-home admittance or limiting the involvement of outside carers.

For peer review only

Discussion

This paper covers unique and underexplored topics in a COVID-19 vulnerable group. Focussed research on the needs of persons or groups who are often disadvantaged in an emergency health situation are very much needed. There is limited work with these groups in the UK, and this is especially true in COVID-19. Themes were wide-ranging and identified a number of ways that the COVID-19 pandemic has impacted BAME persons with dementia and carers with regards to their experiences of dementia community care, and impact on their everyday lives. The results showed that such impacts were far-reaching and affected not only day-to-day concerns but also care decisions with long-ranging consequences, and existential interests around fear, faith, death and identity.

Support, or a lack thereof

BAME groups have been disproportionately affected by the COVID-19 pandemic in the UK [18]. In conjunction with this, past meta-analysis has identified significant barriers faced by members of BAME groups in accessing dementia care [5] both at the community and the service level. Many of our respondents, both persons with dementia and carers, spoke about their own experiences of help-seeking - whether in light of a lack of available services, or difficulty in the process of seeking them. Uncertainty around service provision, a lack of access without pre-pandemic relationships with services and practical difficulties in accessing support digitally were all sources of anxiety. Some patients and caregivers will not have these relationships as the pandemic has changed their needs, and thus required support levels. Independently of COVID-19, some people's needs will also naturally have changed due to decline over time.

Both persons with dementia and carers remarked on feeling great loss at a lack of routine and identity outside of the caring relationship. Past research [19] has shown strong correlation between carer burden and subsequent depression and wellbeing. This has been shown to be related to use of outside sources of support. In a circumstance where outside support has been found to be difficult to access across the board, particular attention should be paid to the burden of burnout and depression in carers moving forwards. Similarly, in persons with dementia independence and autonomy have been posited as key in maintaining positive identities in bound circumstances - namely care homes [20]. These will have been affected by COVID-19 on both micro and macro levels, from government regulations to changes to routines made on the family level in response to fear and anxiety.

A key form of support that was specifically probed in interviews, was behaviour around food and eating. Food insecurity throughout the pandemic has been widespread, and related to external factors such as employment security, stability of housing and isolation. Barker and Russell [21] identify a number of groups who were particularly at risk of food insecurity under the first COVID-19 lockdown, of which our respondents typically occupy at least two – namely being members of minority populations, and having or being a carer for somebody with a chronic illness. Thus, many respondents had been recipients of government-supplied food parcels, which were standardised and tailored towards a typically White British diet.

For peer review only - http://bmjopen.bmj.com/site/about/guidelines.xhtml

Food and eating behaviours are inextricably tied-up with ethnic and cultural identity, and the lack of availability of culturally appropriate food and ingredients during the first COVID-19 lockdown was the source of some distress for a number of interviewees.

This concern was also echoed in reference to community support and social interaction. Many reported a lack of being able to engage with usual forms of rites of passage, cultural milestones or celebrations with their community, and this in-turn added to feelings of isolation and lack of identity. Often, attendance at rites of passage – particularly funerals – signifies a person's social standing within the community. Thus the impact of having a sparsely-attended funeral is upsetting for those left behind and may contribute to compounded and complex grief [22]

These factors, in conjunction, constitute a form of double-disadvantaging whereby members of BAME communities living with or caring for those with dementia are not only disproportionately affected by the COVID-19 pandemic through systemic deprivation, but also by known barriers to service access, support or disenfranchisement through lack of access to culturally and ethnically appropriate provision, genetic factors and overrepresentation in frontline occupations.

Making plans and decisions

The majority of respondents, both persons with dementia and carers, had not made key medical or care decisions in advance of the COVID-19 pandemic. Previous research suggests that there are several reasons for such conversations not taking place in advance, from an expectation that family members will decide what is best [23], to a lack of tailored interventions that positively promote advance decision-making in minority groups [24]. Many carers reported feeling overwhelmed by information in the media about the level of risk, and potential lack of provision, for older people regarding medical care. A continued cause for concern was the restriction of visitors in acute medical and tertiary care settings. Several interviewees, both persons with dementia and carers, remarked that medical interactions had been especially stressful in light of a person with diminished capacity not having a family member to help them communicate and advocate in the medical setting. A COVID-19 prompted rapid-review of evidence around decision-making in older adults [25] identified the need for interventions to be culturally tailored, and that several studies highlighted a lack of positive response among BAME communities to generic planning interventions. Data from the interviews in this paper support these findings - from differing familial roles in decision-making to the need to recognise culturally specific ideas around autonomy and "what is proper".

Adapting to COVID-19

Adapting to new restrictions and ways of living under the first COVID-19 lockdown in the UK proved to be challenging for many, but those living with dementia and those involved in caring for persons with dementia faced extra challenges around care provision, help-seeking and lack of service availability. This in some cases is also compounded by difficulties in learning the skills required to access services remotely. Geibel et al [26]identified a significant reduction in support service usage amongst a population of persons with

For peer review only - http://bmjopen.bmj.com/site/about/guidelines.xhtml

dementia and their carers in the general population. This has potential significant future effects on distress and mental wellbeing levels in both persons with dementia and carers. Burden of behavioural and psychological symptoms of dementia (BPSD) have been consistently found to be associated with an increased risk of nursing home admission [27]. Thus, the increase in those dealing with BPSD in persons with dementia under lockdown may prove to have significant repercussions on public health through potential increase in future care home admissions.

Technology was used to access a huge range of services and interactions within our respondent group, from healthcare to social and religious ceremonies. As our respondents in a number of cases were recruited from an online dementia research registry, our sample may skew more tech-savvy than most. Many outreach groups strongly promoted digital literacy training during the pandemic, which may also have had a positive effect. However, those without the capacity to access digital services may end up further disadvantaged. Previous literature [28,29] has highlighted the importance of enabling older adults with dementia and carers to use technology to promote social interaction, and the unique circumstances of the COVID-19 pandemic may have hastened the need for widespread digital inclusion – both for social needs and wider functions such as maintaining contact with those in hospitals and care homes, attending telehealth appointments and ordering groceries.

COVID-19 and dementia

Emerging evidence suggests that the impact of COVID-19 on persons living with dementia and their families has highlight specific challenges and support needs. Many are concerned that the effects of lockdown – lack of social contact, decreased cognitive stimulation and a lack of outside purpose – may have a lasting and deleterious effect on those with dementia and other cognitive impairments [30]. These findings were also echoed in the experiences of our respondents. Studies that have measured the psychological impact of pandemic in this population have found increased levels of depression, anxiety and loneliness [31], which were particularly related to a lack of understanding and knowledge about the pandemic, and needs for lockdown. A national longitudinal survey of support service usage and mental wellbeing in persons living with dementia and their families [32] found a significant drop in usage shortly after lockdown was enacted. This is an experience shared by the participants in this study, who expressed many difficulties, fears and anxieties in accessing services during this time. This study adds a valuable BAME-specific point of view to current literature.

Strengths and Limitations

This paper covers underexplored topics at-risk groups during COVID-19. BAME groups are both particularly vulnerable and understudied in this context. However, there are certain limitations to our approach. This study focussed on South Asian and Afro-Caribbean groups, and views may not be generalisable to other minority groups. However, the spirit of the findings – i.e. ensuring cultural appropriateness in services and interventions – is widely applicable. We also had a likely self-selecting tech-savvy study population. Due to some aspects of recruitment (via online registries) and the mode of study (interview via Zoom or

For peer review only - http://bmjopen.bmj.com/site/about/guidelines.xhtml

other video call) we reached people who already had a decent level of access to technology, and/or could be facilitated in this by an informal carer. This is not widely applicable to all older adults.

In qualitative research, the role and positioning of the researcher naturally has an impact on the research performed, in both conducting and analysing research. The researchers who performed the interviews were of differing professions and ethnic backgrounds, which may have affected the way that participants responded to them – especially in cases where participants were of the same background as the interviewer, and so assumed knowledge or understanding of the cultural situation. A strength of our approach is the diversity of the research team. That similar findings came out of interviews conducted by each researcher shows that the questions used and the approach taken elicited these responses, rather than identification with the interviewer. Similarly, a diverse research team checked and ratified the analysis, to minimise bias or oversight based on background or personal experience.

Conclusions

BAME communities, including persons with dementia and their carers, in the UK have been heavily impacted by the COVID-19 pandemic. These impacts have been multi-faceted, beginning with susceptibility to the virus and encompassing aspects of life as diverse and service access, social relationships and access to culturally specific resources. Underpinning many of these experiences has been a paucity of support. There are potentially long-reaching effects of these experiences that will be uncovered as the pandemic continues and beyond, including increased support needs of patients and carers, looking at promoting equity of service access and provision and exploring the role of technology in health and social care in the future. This paucity of support should be addressed to prevent potential widening inequalities and negative impacts on health and wellbeing.

Conflicts of Interest

The authors named certify that they have NO affiliations with, or involvement in, any organization or entity with any financial or non-financial interest in the subject matter or materials discussed in this manuscript.

Author Statement

PN performed the majority of interviews and developed the coding framework. EW performed extra interviews, contributed to the coding framework, performed analysis and drafted the manuscript. YBM refined the coding framework and contributed to the manuscript. KW, NK and ES contributed to the manuscript. ND contributed to the manuscript and provided adjudication on analysis. Thanks to our patient and public involvement panel for providing valuable input into study design and strategy.

For peer review only - http://bmjopen.bmj.com/site/about/guidelines.xhtml

Bibliography

1. Alzheimer, Society. *Dementia UK Second Edition - Overview*., 2007.
2. Improving Dementia Services in England: An Interim Report - Great Britain. National Audit Office - Google Books.
3. Cooper C, Tandy AR, Balamurali TBS *et al.* A systematic review and meta-analysis of ethnic differences in use of dementia treatment, care, and research. *Am J Geriatr Psychiatry* 2010;**18**:193–203.
4. Daker-White G, Beattie AM, Gilliard J *et al.* Minority ethnic groups in dementia care: A review of service needs, service provision and models of good practice. *Aging Ment Heal* 2002;**6**:101–8.
5. Kenning C, Daker-White G, Blakemore A *et al.* Barriers and facilitators in accessing dementia care by ethnic minority groups: A meta-synthesis of qualitative studies. *BMC Psychiatry* 2017;**17**:1–13.
6. Mukadam N, Cooper C, Livingston G. A systematic review of ethnicity and pathways to care in dementia. *Int J Geriatr Psychiatry* 2011;**26**:12–20.
7. Pham TM, Petersen I, Walters K *et al.* Trends in dementia diagnosis rates in uk ethnic groups: Analysis of uk primary care data. *Clin Epidemiol* 2018;**10**:949–60.
8. Adelman S, Blanchard M, Livingston G. A systematic review of the prevalence and covariates of dementia or relative cognitive impairment in the older African-Caribbean population in Britain. *Int J Geriatr Psychiatry* 2009;**24**:657–65.
9. Giebel CM, Zubair M, Jolley D *et al.* South Asian older adults with memory impairment: improving assessment and access to dementia care. *Int J Geriatr Psychiatry* 2015;**30**:345–56.
10. *Homecare and Dementia Dementia and Homecare: Driving Quality and Innovation*., 2015.
11. Lewis F, Karlsberg Schaffer S, Sussex J *et al. The Trajectory of Dementia in the UK-Making a Difference*., 2014.
12. Suárez-González A, Livingston G, Low LF *et al.* Impact and Mortality of COVID-19 on People Living With Dementia: Cross-Country Report. *Inst Aging Publ* 2020.
13. Razaq A, Harrison D, Barr B *et al.* BAME Covid-19 Deaths - what do we know?: rapid data & evidence review. 2020.
14. *Disparities in the Risk and Outcomes of COVID-19*., 2020.
15. Bell S, Clarke R, Mounier-Jack S *et al.* Parents' and guardians' views on the acceptability of a future COVID-19 vaccine: A multi-methods study in England. *Vaccine* 2020;**38**:7789–98.
16. Paul E, Steptoe A, Fancourt D. Attitudes towards vaccines and intention to vaccinate against COVID-19: Implications for public health communications. *Lancet Reg Heal - Eur* 2020:100012.
17. The Constructivist Credo - Yvonna S Lincoln, Egon G Guba - Google Books.
18. Rose T, Mason K, Pennington A *et al.* Inequalities in COVID19 mortality related to ethnicity and socioeconomic deprivation. *medRxiv Prepr Serv Heal Sci* 2020:2020.04.25.20079491.
19. Miller VJ, Killian MO, Fields N. Caregiver identity theory and predictors of burden and depression: Findings from the REACH II study. *Aging Ment Health* 2020;**24**:212–20.
20. Paddock K, Brown Wilson C, Walshe C *et al.* Care Home Life and Identity: A Qualitative Case Study. *Gerontologist* 2019;**59**:655–64.
21. Barker M, Russell J. Feeding the food insecure in Britain: learning from the 2020 COVID-

For peer review only - http://bmjopen.bmj.com/site/about/guidelines.xhtml

19 crisis. *Food Secur* 2020;**12**:865–70.
22. Kokou-Kpolou CK, Fernández-Alcántara M, Cénat JM. Prolonged Grief Related to COVID-19 Deaths: Do We Have to Fear a Steep Rise in Traumatic and Disenfranchised Griefs? *Psychol Trauma Theory, Res Pract Policy* 2020;**12**:S94.
23. De Souza J, Gillett K, Froggatt K *et al.* Perspectives of elders and their adult children of Black and minority ethnic heritage on end-of-life conversations: A meta-ethnography. *Palliat Med* 2020;**34**:195–208.
24. Kwak J, Haley WE. Current Research Findings on End-of-Life Decision Making Among Racially or Ethnically Diverse Groups. *Gerontologist* 2005;**45**:634–41.
25. West E, Moore K, Kupeli N *et al.* Rapid review of decision-making for place of care and death in older people: lessons for COVID-19. *Age Ageing* 2020:1–13.
26. Giebel C, Cannon J, Hanna K *et al.* Impact of COVID-19 related social support service closures on people with dementia and unpaid carers: a qualitative study. *Aging Ment Health* 2020:1–8.
27. Toot S, Swinson T, Devine M *et al.* Causes of nursing home placement for older people with dementia: A systematic review and meta-analysis. *Int Psychogeriatrics* 2017;**29**:195–208.
28. Davies N, Iliffe S, Hopwood J *et al.* The key aspects of online support that older family carers of people with dementia want at the end of life: A qualitative study. *Aging Ment Heal* 2020;**24**:1654–61.
29. Holthe T, Halvorsrud L, Karterud D *et al.* Usability and acceptability of technology for community-dwelling older adults with mild cognitive impairment and dementia: A systematic literature review. *Clin Interv Aging* 2018;**13**:863–86.
30. Talbot C V, Briggs P. “Getting back to normality seems as big of a step as going into lockdown”: The Impact of the COVID-19 Pandemic on People with Early-Middle Stage Dementia. *Age Ageing* 2021:1–7.
31. Thyrian JR, Kracht F, Nikelski A *et al.* The situation of elderly with cognitive impairment living at home during lockdown in the Corona-pandemic in Germany. *BMC Geriatr* 2020;**20**:540.
32. Giebel C, Pulford D, Cooper C *et al.* COVID-19-related social support service closures and mental well-being in older adults and those affected by dementia: A UK longitudinal survey. *BMJ Open* 2021;**11**:45889.

For peer review only

For peer review only - http://bmjopen.bmj.com/site/about/guidelines.xhtml

Topic Guide - An Exploration of the impact of Covid-19 on dementia care in Black and Minority Ethnic groups (Carers)

Introduction

- Thank you for agreeing to take part in this research study.
- This study aims to meet the needs of people living with dementia and support those close to them (family and friends) such as yourself during the Corona Virus pandemic. We aim to understand how carers and people with dementia have been affected by the current pandemic. Importantly we would like to find out if and how experiences related to dementia care, services, caring and food and drink have been affected.
- Before we start can I just check you have had chance to read through the information sheet?
- Do you have any questions about it?
- Do you agree to be audio recorded?
- Reiterate consent has been taken and happy to proceed

Check recording device on!

1. **Can you tell me who it is you care for and where they are currently living? What is your daily routine like?**
2. **How has the situation with regards to Coronavirus impacted on how you are able to provide care or support your relative/friend with dementia?** (e.g. daily routines, care home context)
    a. Have you had any particular challenges or challenging moments?

For peer review only

b. Have you explained to (name of person with dementia) what's going on? What information have they been given?
c. Respite services and home care workers – are they are wearing PPE? How has this affected the PwD (e.g. are they reacting distressed etc)
d. Have you had to make any decisions about their care? If so, why and how?
e. Do you feel more or less worried about the coronavirus situation in view of your ethnic background? (reports of higher incidence..)
f. *If relative in a care home*: have you been able to visit your family member with dementia? Have you received sufficient communication from the care home?
g. Explore socio-cultural impact, e.g. birthdays festivals
h. Do you know anyone who has been affected by corona virus? (bereavements, able to attend funerals, visiting)

3. Thinking about the person you provide care and support for, how has (name of person with dementia) reacted to the corona virus situation?

a. Has it had an effect on them do you think? (explore impact of news/information etc)
b. How has it affected their wellbeing? What, if anything, has helped maintain their wellbeing during this situation?
c. Have their needs changed?
d. Probe about routine and how this has been affected
e. What have you found is helpful to them?
f. Explore role of technologies for keeping in touch, e.g. telephone, video

4. Could you describe if and how Corona virus impacts your current situation regarding access to/use of dementia care services and support?

- How has it affected your access to the GP?
- How has it affected your access to community dementia care services, e.g. dementia hubs/organisations, memory clinic, carer organisations?
- How have you been able to communicate with healthcare teams? How do you feel about this?

For peer review only

- Have you had any conversations around advance care planning in view of Covid (i.e. if PwD becomes unwell)?

5. **Now thinking about your needs as a carer. Could you describe if/how the Corona virus has impacted on your current needs and situation?**
   - How has it affected your caring arrangements? (e.g. family or formal carers/social care)
   - How has it affected your social life / family life?
   - How has it affected your wellbeing?
   - Do you think the effect for you is heightened, as a carer of someone with dementia from a BAME background?
   - What do you think about the way in which the coronavirus pandemic has been reported by the government/news? How has it affected you?
   - Has the government advice clear or confusing?
   - What extra support, if any, have you had/been offered? Has this support been appropriate for your specific/cultural needs? (e.g. voluntary services, community support, formal support)
   - What has helped you to maintain your wellbeing as a carer during the Corona virus situation?

6. **What kind of support would you have liked to have had that are not available due to Corona virus? (**e.g. help from friends/family, help from professionals, help with family tasks like shopping, emotional/psychological support)

7. **How does the situation with regard to Corona virus impact on how you are able to support (name of person with dementia) to continue eating and drinking?**

- Can you tell me a bit about your experience of managing meals and food for XX?
- Have you noticed any changes in their eating and drinking recently? Has the Coronavirus situation impacted on this (e.g. stress, anxiety, changes to routine)

For peer review only

- ➢ If they are in a care home – how has their management of food and drink been affected? Are they usually involved in their diet? If so, how?
- ➢ Do you think food has different roles in different cultures? (explore religious significance, festivals)
- ➢ Is the social/communal aspect of food important to you and for the PwD? (e.g. family mealtimes)
- ➢ How has COVID-19 affected shopping for food?
- ➢ How does it affect food deliveries? Food parcels?
- ➢ How does it affect availability of certain foods? E.g. cultural foods
- ➢ How have you managed this? Have you received any help/support?
- ➢ Has it caused you to change their diet or how you prepare their food? If so, how? What impact has this had? And how do you manage this?
- ➢ Has it affected your mealtimes? E.g. family meals? How has social distancing impacted on this?
- ➢ Have any cultural events/festivals been affected (e.g. Ramadan)? If so, in what way?
- ➢ What support are you currently receiving with regards to helping you manage food and drink? Is there any other support you would like? In what format/delivery?

Close

- Check if the participant would like to add anything not already covered
- Summarise some take home messages from the interview
- Thank the participant for their time today
- Stop recording
- **Complete demographic checklist**
- **Post gift voucher.**

For peer review only

An Exploration of the impact of Covid-19 on dementia care in Black and Minority Ethnic groups (People with dementia)

Introduction

- Thank you for agreeing to take part in this research study.

- This study aims to meet the needs of people living with dementia such as yourself and support those close to them (family and friends during the Corona Virus pandemic. We aim to understand how carers and people with dementia have been affected by the current pandemic. Importantly we would like to find out if and how experiences related to dementia care, services, caring and food and drink have been affected.

- Before we start can I just check you have had chance to read through the information sheet?

- Do you have any questions about it?

- Do you agree to be audio recorded?

- Reiterate consent has been taken and happy to proceed.

Check recording device on!

8. How are you//how has your week been? Just to start off – can you tell me what your typical routine/day looks like?

9. **How does the situation with regard to Coronavirus impact your daily life? (e.g. daily routines)**
   - How has it affected you daily routine? (ask if in a care home, how it has affected daily life….)
   - How has it affected your social/family life? (e.g. birthdays, festivals)

For peer review only

- Have you had any particularly challenging moments?
- How has it affected your wellbeing?
- Have your needs changed?
- Do you think Coronavirus has a greater impact on you, as someone experiencing memory problems from an ethnic minority background?
- What extra support, if any, have you been offered?
- What has helped you maintain your wellbeing during this time?
- Do you know anyone who has been affected by coronavirus (bereavements, able to attend funerals, visiting)
- What do you think about the way in which the coronavirus pandemic has been reported by the government/news? How has it affected you?

10. **Could you describe if and how Corona virus impacts your current situation regarding access to/use of dementia care services and support?**
    - How has it affected your access to the GP?
    - How has it affected your access to community dementia care services, e.g. dementia hubs/organisations, memory clinic?
    - Has it affected your formal caring (if any) arrangements?
    - How have you been able to communicate with healthcare teams? How do you feel about this?
    - Have you had any discussions about your care during this time and planning for the future (e.g. if you become unwell/advance care planning)?
    - What extra support, if any, have you had/been offered? Has this support been appropriate for your specific/cultural needs? (e.g. voluntary services, community support, formal support)

11. **What kind of support would you have liked to have had that are not available due to Corona virus?** (e.g. help from friends/family, help from professionals, help with family tasks like shopping, emotional/psychological support)

12. **Specific questions about eating and drinking in dementia:**

For peer review only

- Since your diagnosis, have you noticed any changes in your eating/drinking? (e.g. flavours, portions, textures; mealtimes)
- What do you think the role of food has in your culture/life? Does food/drink have any religious significance for you? Festivals?
- Is the social/communal aspect of food important to you? (e.g. family mealtimes)

13. How does the situation with regard to Corona virus impact on your eating and drinking needs?

  - How does it affect shopping for food? How has this affected you?
  - How does it affect food deliveries? Food parcels?
  - How does it affect availability of food? E.g. cultural foods
  - Have you received any help/support with this? (e.g. voluntary, community or formal support)
  - Has it caused you to change your diet? If so, how? Has this had an impact on your health?
  - Has it affected your mealtimes? E.g. social interactions with others around mealtimes/ family meals? How has social distancing impacted on this?
  - Have any cultural events/festivals been affected? If so, in what way?
  - What support are you currently receiving with regards to helping you manage food and drink? Is there any other support you would like? In what format/delivery?

Close

- Check if the participant would like to add anything not already covered
- Summarise some take home messages from the interview
- Thank the participant for their time today
- Stop recording
- **Complete demographic checklist**
- **Post gift voucher.**

For peer review only

For peer review only

COREQ (COnsolidated criteria for REporting Qualitative research) Checklist

A checklist of items that should be included in reports of qualitative research. You must report the page number in your manuscript where you consider each of the items listed in this checklist. If you have not included this information, either revise your manuscript accordingly before submitting or note N/A.

Topic	Item No.	Guide Questions/Description	Reported on Page No.
**Domain 1: Research team and reflexivity**			
Personal characteristics			
Interviewer/facilitator	1	Which author/s conducted the interview or focus group?	
Credentials	2	What were the researcher's credentials? E.g. PhD, MD	
Occupation	3	What was their occupation at the time of the study?	
Gender	4	Was the researcher male or female?	
Experience and training	5	What experience or training did the researcher have?	
Relationship with participants			
Relationship established	6	Was a relationship established prior to study commencement?	
Participant knowledge of the interviewer	7	What did the participants know about the researcher? e.g. personal goals, reasons for doing the research	
Interviewer characteristics	8	What characteristics were reported about the inter viewer/facilitator? e.g. Bias, assumptions, reasons and interests in the research topic	
**Domain 2: Study design**			
Theoretical framework			
Methodological orientation and Theory	9	What methodological orientation was stated to underpin the study? e.g. grounded theory, discourse analysis, ethnography, phenomenology, content analysis	
Participant selection			
Sampling	10	How were participants selected? e.g. purposive, convenience, consecutive, snowball	
Method of approach	11	How were participants approached? e.g. face-to-face, telephone, mail, email	
Sample size	12	How many participants were in the study?	
Non-participation	13	How many people refused to participate or dropped out? Reasons?	
Setting			
Setting of data collection	14	Where was the data collected? e.g. home, clinic, workplace	
Presence of non-participants	15	Was anyone else present besides the participants and researchers?	
Description of sample	16	What are the important characteristics of the sample? e.g. demographic data, date	
Data collection			
Interview guide	17	Were questions, prompts, guides provided by the authors? Was it pilot tested?	
Repeat interviews	18	Were repeat inter views carried out? If yes, how many?	
Audio/visual recording	19	Did the research use audio or visual recording to collect the data?	
Field notes	20	Were field notes made during and/or after the inter view or focus group?	
Duration	21	What was the duration of the inter views or focus group?	
Data saturation	22	Was data saturation discussed?	
Transcripts returned	23	Were transcripts returned to participants for comment and/or	

For peer review only

Topic	Item No.	Guide Questions/Description	Reported on Page No.
		correction?	
**Domain 3: analysis and findings**			
Data analysis			
Number of data coders	24	How many data coders coded the data?	
Description of the coding tree	25	Did authors provide a description of the coding tree?	
Derivation of themes	26	Were themes identified in advance or derived from the data?	
Software	27	What software, if applicable, was used to manage the data?	
Participant checking	28	Did participants provide feedback on the findings?	
Reporting			
Quotations presented	29	Were participant quotations presented to illustrate the themes/findings? Was each quotation identified? e.g. participant number	
Data and findings consistent	30	Was there consistency between the data presented and the findings?	
Clarity of major themes	31	Were major themes clearly presented in the findings?	
Clarity of minor themes	32	Is there a description of diverse cases or discussion of minor themes?	

Developed from: Tong A, Sainsbury P, Craig J. Consolidated criteria for reporting qualitative research (COREQ): a 32-item checklist for interviews and focus groups. *International Journal for Quality in Health Care*. 2007. Volume 19, Number 6: pp. 349 – 357

Once you have completed this checklist, please save a copy and upload it as part of your submission. DO NOT include this checklist as part of the main manuscript document. It must be uploaded as a separate file.

For peer review only
